# Metastatic Extrapulmonary Small Cell Carcinoma to the Cerebellopontine Angle: A Case Report and Review of the Literature

**DOI:** 10.1155/2015/847058

**Published:** 2015-02-25

**Authors:** Debebe Theodros, C. Rory Goodwin, Genevieve M. Crane, Jason Liauw, Lawrence Kleinberg, Michael Lim

**Affiliations:** ^1^Department of Neurosurgery, The Johns Hopkins Hospital, The Johns Hopkins University School of Medicine, Baltimore, MD 21287, USA; ^2^Department of Pathology, The Johns Hopkins Hospital, The Johns Hopkins University School of Medicine, Baltimore, MD 21287, USA; ^3^Department of Radiation Oncology, The Johns Hopkins Hospital, The Johns Hopkins University School of Medicine, Baltimore, MD 21287, USA

## Abstract

Extrapulmonary small cell carcinomas (EPSCC) are rare malignancies with poor patient prognoses. We present the case of a 63-year-old male who underwent surgical resection of a poorly differentiated small cell carcinoma, likely from a small intestinal primary tumor that metastasized to the cerebellopontine angle (CPA). A 63-year-old male presented with mild left facial paralysis, hearing loss, and balance instability. MRI revealed a 15 mm mass in the left CPA involving the internal auditory canal consistent with a vestibular schwannoma. Preoperative MRI eight weeks later demonstrated marked enlargement to 35 mm. The patient underwent a suboccipital craniectomy and the mass was grossly different visually and in consistency from a standard vestibular schwannoma. The final pathology revealed a poorly differentiated small cell carcinoma. Postoperative PET scan identified avid uptake in the small intestine suggestive of either a small intestinal primary tumor or additional metastatic disease. The patient underwent whole brain radiation therapy and chemotherapy and at last follow-up demonstrated improvement in his symptoms. Surgical resection and radiotherapy are potential treatment options to improve survival in patients diagnosed with NET brain metastases. We present the first documented case of skull base metastasis of a poorly differentiated small cell carcinoma involving the CPA.

## 1. Introduction

Poorly differentiated extrapulmonary small cell carcinoma (EPSCC) is a particularly rare subtype of neuroendocrine carcinoma that accounts for 0.1–1.0% of gastrointestinal (GI) tumors. Furthermore, the small intestine represents a rare primary site of EPSCC accounting for 0.2% of all GI malignancies [1–3]. The median age of diagnosis for EPSCC is 70 years with more than 50% of patients presenting with regional lymph node involvement or distant metastasis [3–10]. Multiple case series and single-institution experiences within the literature have attempted to capture the disease course and prognostic factors associated with patients with EPSCC. The median time to progression for patients with limited disease (LD) or extensive disease (ED) is approximately 20 months and 12 months, respectively [11, 12]. The overall survival for patients with EPSCC has been shown to range from 2 to 43 months [11–14]. Despite a dismal prognosis, multimodal therapy improves 2-year survival from 42% to 83% and 5-year survival from 21% to 63% in comparison to local or systemic therapy alone [12].

Brain metastasis is rare and predicts a poor outcome. Overall survival from time of metastasis diagnosis to death varies considerably, from 1 week to 22 months, with some patients showing modest responses to radiotherapy [15–20]. Most patients ultimately present with neurologic symptoms, as well as multiple metastasis to the brain [15–22]. In this paper, we describe the first documented case to the knowledge of the authors of a 63-year-old patient who presented with disturbances of the left 3rd through 8th cranial nerves and underwent surgical resection for skull base metastasis of a poorly differentiated small cell tumor metastasis to the cerebellopontine angle (CPA). We also review the literature on EPSCC with a focus on metastasis to the brain.

## 2. Case Presentation

**Figure 1:**
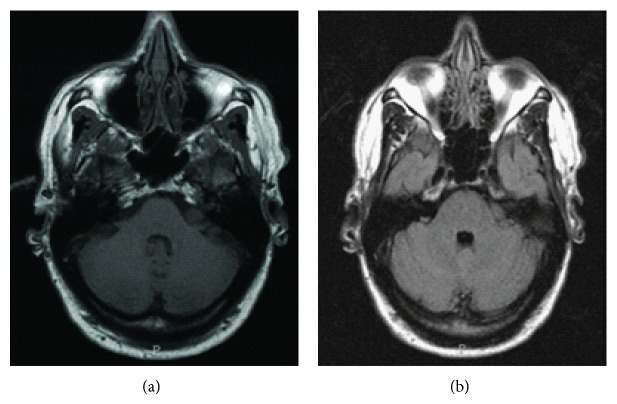
(a) T1-axial MRI with contrast and (b) T2-axial Flair MRI performed at initial encounter demonstrate a minimally enhancing mass in the left cerebellopontine angle.

**Figure 2:**
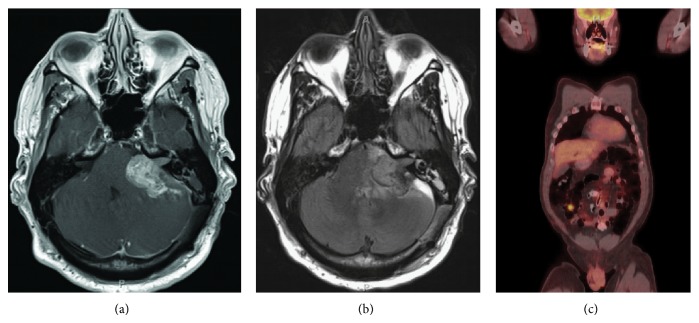
(a) T1-axial MRI with contrast and (b) T2-axial Flair MRI demonstrating enhancing mass in the left cerebellopontine angle 8 weeks later. (c) Post-operative whole body positron emission tomography demonstrating increased uptake in the right lower quadrant corresponding to the small intestine.

**Figure 3:**
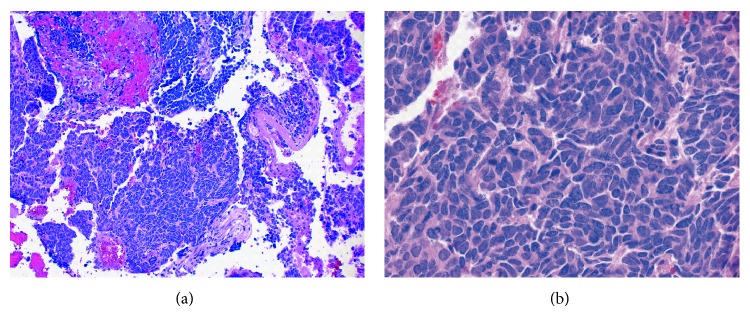
Pathology of the fragmented CPA tumor mass demonstrated poorly differentiated neuroendocrine carcinoma (small cell). (a) The tumor was highly cellular with tumor cells demonstrating a high nuclear to cytoplasmic ratio and molded nuclei (64x). (b) Higher magnification revealed characteristic finely granular, “salt and pepper” chromatin and frequent mitoses (254x). Both photomicrographs are of an H&E stained section. A Ki-67 proliferation index was high, and neoplastic cells were positive for cytokeratin, synaptophysin, and CD56 (not shown).

**Figure 4:**
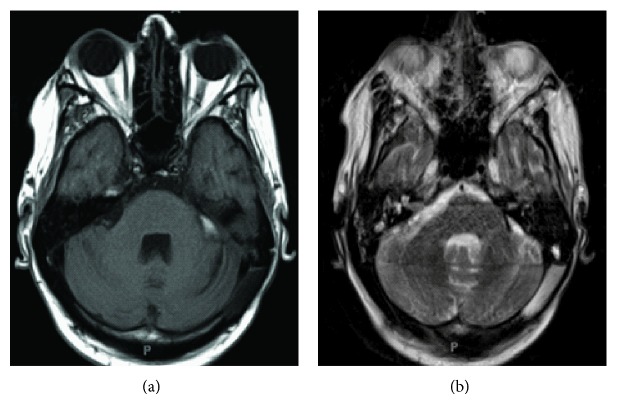
(a) T1-axial MRI with contrast and (b) T2-axial Flair MRI demonstrating interval decrease in size of previously identified enhancing mass in the left cerebellopontine angle after resection.

A 63-year-old man with a past medical history of hypertension, hyperlipidemia, hypothyroidism, and gout presented with left facial paralysis, dryness and irritation in his left eye, hearing loss, and balance instability that occurred over the course of weeks. The patient was initially diagnosed with benign paroxysmal positional vertigo and started on meclizine with no relief. He was referred to an otolaryngologist and magnetic resonance imaging (MRI) was performed at an outside institution that demonstrated a 15 mm enhancing mass in the left CPA with involvement of the internal auditory canal that was most consistent with a vestibular schwannoma. The patient was counseled as to the different treatment options for a presumed vestibular schwannoma with repeat imaging performed at our institution and chose surgery (Figure 1). Eight weeks later, the patient began experiencing worsening facial function, was classified as a House-Brackmann VI on physical examination, and also reported difficulty with swallowing. A repeat MRI revealed the mass had increased to 35 mm with mass effect on the pons, superior, middle, and inferior cerebellar peduncles with associated edema (Figure 2). The patient subsequently underwent a left-sided retrosigmoid craniotomy. Intraoperatively, the dura and the tumor tissue itself were erythematous, friable, and extremely hemorrhagic. The mass also had an unexpected consistency and appearance as it was very soft, friable, and purplish in color. Preliminary frozen specimens sent to pathology were concerning for lymphoma. The patient's tumor was then debulked. Post-operatively, the patient underwent a whole body PET scan, which demonstrated high FDG activity in the location of the known residual left CPA mass, as well as an additional hypermetabolic mesenteric lesion abutting the ileum and ascending colon in the right lower quadrant (Figure 2). The final pathology report identified the CPA lesion as a poorly differentiated small cell neuroendocrine carcinoma and was confirmed by positive immunostaining of the tumor cells for cytokeratin CAM 5.2, synaptophysin, and CD56 and a high proliferative index (Figure 3). The tumor cells were negative for CD3, CD20, Epstein-Barr virus, S100, and thyroid transcription factor-1 (TTF-1). The mesenteric lesion was not biopsied at this time. The patient continued to experience symptoms of left facial paralysis, left eye irritation, decreased hearing loss, imbalance, and vertigo postoperatively but was discharged home in good condition. He underwent whole brain radiation therapy followed by chemotherapy and at last follow-up postoperatively has continued to show signs of improvement (Figure 4).

## 3. Discussion

We present the first documented case of skull base metastasis of EPSCC to the CPA. To date, there have been 24 cases of NET brain metastases in the literature, with 3 patients presenting with carcinoid syndrome, 1 with increasing chromogranin A levels, and 1 diagnosed by radiologic imaging (Table 1) [15–22]. The clinical management of patients presenting with a new lesion revolves around confirmation/localization of the disease, staging, and treatment. Serum chromogranin A levels are elevated in 80% of patients with NET regardless of tumor location and correlate with disease burden. More recently, PET/CT with 2-[18F]-fluoro-2-deoxy-D-glucose (FDG) has been used although there is scant evidence investigating the utility of adding this imaging modality for staging to current staging protocols [10, 23, 24]. The role of surgery is complicated in cases of localized and metastatic disease as there is still considerable debate over this issue [1, 7]. Similarly, although GI small cell carcinomas appear to be radiosensitive, cure with radiotherapy is rare; however, concurrent or sequential treatment with chemotherapy may lead to cure or long-term survival in some patients [3, 25–32]. Chemotherapy is shown to induce long-term survival in patients with localized and extended disease with the most commonly used chemotherapeutic drug regimen consisting of cisplatin, etoposide, cyclophosphamide, doxorubicin, and irinotecan [3, 4, 28, 33, 34]. Despite surgical resection and multimodal adjuvant therapy, the prognosis is generally poor [9].

Given our patient's presentation, no prior history of cancer and initial imaging findings, vestibular schwannoma was at the top of our differential diagnosis. However, given the rapid expansion of the extra-axial mass, we reevaluated our differential, as this was atypical for vestibular schwannomas. Given the mass effect and early hydrocephalus, we elected to proceed with surgical resection, given this treatment modality would allow for diagnosis and debulking of the tumor. Increased serum chromogranin A levels and/or 18-FDG PET scans demonstrating avid uptake in the small intestine performed at an earlier stage would have suggested a different diagnosis of metastatic disease. However, our treatment paradigm would have been the same in both situations with surgical resection, whole brain radiation therapy, and chemotherapy, similar to other reports for brain metastasis in the literature. While a small intestinal primary tumor cannot be definitively shown in this case, the FDG avidity of the mesenteric mass combined with the tumor cells being TTF-1 negative strongly suggests a gastrointestinal primary. While TTF-1 positivity can be seen in both pulmonary and extrapulmonary small cell carcinomas, TTF-1 was not observed in one large series evaluating metastatic neuroendocrine neoplasms of intestinal origin [35, 36].

This case represents the first documented case of skull base metastasis of EPSCC and presented several treatment challenges. The role of chemotherapy versus radiation therapy versus surgical therapy is unclear in patients with skull base metastasis and each is not without its significant risks. The indications for the various treatments may also differ based on site of metastasis; however, this piece of information is currently unknown. Furthermore, the prognosis of patients with skull base metastasis is unclear, which would also help guide treatment options. The metastatic tissue in this case was not amenable to* en bloc* surgical resection; however, piecemeal surgical resection with subsequent radiation therapy and chemotherapy ultimately achieved improved function.

We present the first documented case of a patient presenting with skull base metastasis to the CPA of GI EPSCC. Similar to Isaka et al. findings, the tumor was found to be highly vascular posing great difficulty in establishing hemostasis [17]. Brain metastasis of EPSCC may be more common than originally thought, as patients may initially present with symptoms related to brain metastasis and not of the primary tumor. Unfortunately, the majority of patients will present with disease not confined to the primary tumor site, leading to poor prognoses. Enhanced imaging modalities and the use of radiotherapy with concurrent or sequential chemotherapy may provide a survival benefit; however, further work is necessary to help define the optimal treatment options for patients with metastatic EPSCC disease to the brain.

## Figures and Tables

**Table 1:** 

Authors	Patient sex	Age	Presenting symptoms	Primary site	Imaging identification of metastasis	Metastasis site	Treatment	Outcome
Tewari et al. [19]	Female	47	Rising serum chromogranin A on follow-up	Gallbladder	18-FDG PET/CT	Left parietooccipital	RT	Survived

Freedy and Miller [16]	Male	63	Generalized weakness, inappropriate language, jerking movements RUE and RLE	Prostate	Contrast-enhanced CT	Left hemisphere, periventricular	Chemotherapy, palliative measures	Died, 7 days later

Zachariah et al. [20]	Male	72	Confusion, unsteady gait, progressive difficulty talking	Prostate	Contrast-enhanced CT and MRI	Multiple cystic lesions, single right cerebellar hemisphere lesion	WBRT: 30 Gy in 10 treatments	Died, 6 weeks later

Isaka et al. [17]	Male	67	Increasing severity headache	Bladder	CT, T1 and T2, MRI	Cystic lesion left frontal lobe	STR, WBRT: 40 Gy in 20 fractions	Died, 4 months later

Erasmus et al. [15]	Male	70	Left homonymous hemianopsia	Prostate	CT and MRI	Right optic radiation	20 Gy in 5 fractions, booster dose of 15 Gy in 5 doses	Died, 4 months later

Madroszyk et al. [18]	Male	69	General fatigue, 5 kg weight loss	Esophagus	CT	Multiple metastasis	Not discussed	Died, 22 months later
	Male	48	Confusion	Esophagus	CT	Multiple metastasis	RT and corticotherapy	Died, 10 months later

Hussein et al. [21]	Female	50	Vertigo, ataxia, nausea, vomiting	Rectum	CT	Right cerebellar mass	SR, RT (3000 rads)	Survived
	Male	43	Bifrontal headaches, stuporous, right hemiplegia	Colon	CT	Right frontal lobe, left basal ganglion, left subcortical frontal region	SR, RT (3000 rads)	Survived

Mallory et al. [22]	10 M; 5 F	58 (average)	Sensorimotor (6) headache (4), seizures, hydrocephalus, carcinoid syndrome (3)	Lung (7), stomach (4), pancreas (1), large intestine (2), thymus (1), kidney (2), unknown (1)	N/A	Brain	SR (12), GKS (2), WBRT (1)	2 alive at last follow-up, median overall survival 19 months

[15–22].

RUE: right upper extremity, RLE: right lower extremity, RT: radiation therapy, SR: surgical resection, STR: subtotal resection, WBRT: whole brain radiation therapy, and GKS: Gamma Knife surgery.

## References

[1] Brenner B., Tang L. H., Shia J., Klimstra D. S., Kelsen D. P. (2007). Small cell carcinomas of the gastrointestinal tract: clinicopathological features and treatment approach. *Seminars in Oncology*.

[2] Toker C. (1974). Oat cell tumor of the small bowel. *The American Journal of Gastroenterology*.

[3] Brenner B., Tang L. H., Klimstra D. S., Kelsen D. P. (2004). Small-cell carcinomas of the gastrointestinal tract: a review. *Journal of Clinical Oncology*.

[4] Nichols G. L., Kelsen D. P. (1989). Small cell carcinoma of the esophagus. The Memorial Hospital experience 1970 to 1987. *Cancer*.

[5] Bennouna J., Bardet E., Deguiral P., Douillard J. Y. (2000). Small cell carcinoma of the esophagus: analysis of 10 cases and review of the published data. *American Journal of Clinical Oncology*.

[6] Zirkin H. J., Levy J., Katchko L. (1996). Small cell undifferentiated carcinoma of the colon associated with hepatocellular carcinoma in an immunodeficient patient. *Human Pathology*.

[7] Salyers W. J., Vega K. J., Munoz J. C., Trotman B. W., Tanev S. S. (2014). Neuroendocrine tumors of the gastrointestinal tract: case reports and literature review. *World Journal of Gastrointestinal Oncology*.

[8] Vrouvas J., Ash D. V. (1995). Extrapulmonary small cell cancer. *Clinical Oncology*.

[9] Frazier S. R., Kaplan P. A., Loy T. S. (2007). The pathology of extrapulmonary small cell carcinoma. *Seminars in Oncology*.

[10] Joyce E. A., Kavanagh J., Sheehy N., Beddy P., O'Keeffe S. A. (2013). Imaging features of extrapulmonary small cell carcinoma. *Clinical Radiology*.

[11] Haider K., Shahid R. K., Finch D. (2006). Extrapulmonary small cell cancer: a Canadian province's experience. *Cancer*.

[12] Ochsenreither S., Marnitz-Schultze S., Schneider A. (2009). Extrapulmonary small cell carcinoma (EPSCC): 10 Years' multi-disciplinary experience at Charité. *Anticancer Research*.

[13] Kim J. H., Lee S. H., Park J. (2004). Extrapulmonary small-cell carcinoma: a single-institution experience. *Japanese Journal of Clinical Oncology*.

[14] Lee S. S., Lee J. L., Ryu M. H. (2007). Extrapulmonary small cell carcinoma: single center experience with 61 patients. *Acta Oncologica*.

[15] Erasmus C. E., Verhagen W. I., Wauters C. A., van Lindert E. J. (2002). Brain metastasis from prostate small cell carcinoma: not to be neglected. *Canadian Journal of Neurological Sciences*.

[16] Freedy R. M., Miller K. D. (1990). Small-cell carcinoma of the prostate: metastases to the brain as shown by CT and MR with pathologic correlation. *The American Journal of Neuroradiology*.

[17] Isaka T., Maruno M., Sato M. (2002). Brain metastasis from small-cell neuroendocrine carcinoma of the urinary bladder: a case report. *Brain Tumor Pathology*.

[18] Madroszyk A., Egreteau J., Martin L., Queneau P. E., Bosset J. F., Merrouche Y. (2001). Small-cell carcinoma of the esophagus: report of three cases and review of the literature with emphasis on therapy. *Annals of Oncology*.

[19] Tewari A., Palaniswamy S. S., Subramanyam P. (2013). Brain metastasis from neuroendocrine tumor of the gallbladder: a rare entity. *South Asian Journal of Cancer*.

[20] Zachariah B., Casey L., Zachariah S. B., Baekey P., Greenberg H. M. (1994). Case report: brain metastasis from primary small cell carcinoma of the prostate. *The American Journal of the Medical Sciences*.

[21] Hussein A. M., Feun L. G., Sridhar K. S., Otrakji C. L., Garcia-Moore M., Benedetto P. (1990). Small cell carcinoma of the large intestine presenting as central nervous systems signs and symptoms. Two case reports with literature review. *Journal of Neuro-Oncology*.

[22] Mallory G. W., Fang S., Giannini C., van Gompel J. J., Parney I. F. (2013). Brain carcinoid metastases: outcomes and prognostic factors. *Journal of Neurosurgery*.

[23] Fischer B. M., Mortensen J., Langer S. W. (2007). A prospective study of PET/CT in initial staging of small-cell lung cancer: comparison with CT, bone scintigraphy and bone marrow analysis. *Annals of Oncology*.

[24] Vinjamuri M., Craig M., Campbell-Fontaine A., Almubarak M., Gupta N., Rogers J. S. (2008). Can positron emission tomography be used as a staging tool for small-cell lung cancer?. *Clinical Lung Cancer*.

[25] Huncharek M., Muscat J. (1995). Small cell carcinoma of the esophagus: the Massachusetts General Hospital experience, 1978 to 1993. *Chest*.

[26] Medgyesy C. D., Wolff R. A., Putnam J. B., Ajani J. A. (2000). Small cell carcinoma of the esophagus: the University of Texas M. D. Anderson Cancer Center experience and literature review. *Cancer*.

[27] Matsui K., Kitagawa M., Miwa A., Kuroda Y., Tsuji M. (1991). Small cell carcinoma of the stomach: a clinicopathologic study of 17 cases. *The American Journal of Gastroenterology*.

[28] Jereczek-Fossa B., Airoldi M., Vasario E., Ruo Redda M. G., Valente G., Orecchia R. (2000). Small cell carcinoma of the esophagus: a case report and review of the literature. *Tumori*.

[29] Motojima K., Furui J., Terada M. (1990). Small cell carcinoma of the pancreas and biliary tract. *Journal of Surgical Oncology*.

[30] Wahid N. A., Neugut A. I., Hibshoosh H., Brunetti J. C., Fountain K. S., Rubin M. (1996). Response of small cell carcinoma of pancreas to a small cell lung cancer regimen: a case report. *Cancer Investigation*.

[31] Sato Y., Fujisawa J., Saji Y. (1992). A case of small cell undifferentiated carcinoma (SCUC) of the rectum treated with etoposide, cis-platinum and radiotherapy. *Japanese Journal of Cancer and Chemotherapy*.

[32] Casas F., Farrús B., Daniels M. (1996). Six-year follow-up of primary small cell carcinoma of the esophagus showing a complete response: a case report. *Japanese Journal of Clinical Oncology*.

[33] Brenner B., Shah M. A., Gonen M., Klimstra D. S., Shia J., Kelsen D. P. (2004). Small-cell carcinoma of the gastrointestinal tract: a retrospective study of 64 cases. *British Journal of Cancer*.

[34] Casas F., Ferrer F., Farrús B., Casals J., Biete A. (1997). Primary small cell carcinoma of the esophagus: a review of the literature with emphasis on therapy and prognosis. *Cancer*.

[35] Agoff S. N., Lamps L. W., Philip A. T. (2000). Thyroid transcription factor-1 is expressed in extrapulmonary small cell carcinomas but not in other extrapulmonary neuroendocrine tumors. *Modern Pathology*.

[36] Lin X., Saad R. S., Luckasevic T. M., Silverman J. F., Liu Y. (2007). Diagnostic value of CDX-2 and TTF-1 expressions in separating metastatic neuroendocrine neoplasms of unknown origin. *Applied Immunohistochemistry and Molecular Morphology*.

